# Crystal structure of 1-methyl-3-[2,2,2-tri­fluoro-1-(1-methyl-1*H*-indol-3-yl)-1-phenyl­eth­yl]-1*H*-indole

**DOI:** 10.1107/S1600536814021916

**Published:** 2014-10-11

**Authors:** Xian-Rong Liu, Yan-Ling Zhou

**Affiliations:** a School of Chemistry and Chemical Engineering, Guangxi University, Nanning 541004, People’s Republic of China

**Keywords:** crystal structure, 1*H*-indole, tri­fluoro­methyl groups, biological activity, hydrogen bonding

## Abstract

The title compound, C_26_H_21_F_3_N_2_, was prepared by the palladium-catalysed reaction of (2,2,2-tri­fluoro­eth­yl)benzene with 1-methyl-1*H*-indole. The dihedral angle between the planes of the indole-ring systems is 52.13 (6)° and the *N*-methyl groups point away from each other. Three short intra­molecular C—H⋯F contacts are observed.

## Related literature   

For a related structure, see: Zhou *et al.* (2011 ▶). For background to the effect of tri­fluoro­methyl groups, see: Purser *et al.* (2008 ▶). For further synthetic details regarding tri­fluoro­methyl groups, see: Shang *et al.* (2014 ▶); Miura *et al.* (2013 ▶). For background to indole derivatives and their various biological activities, see: Lo *et al.* (2007 ▶).
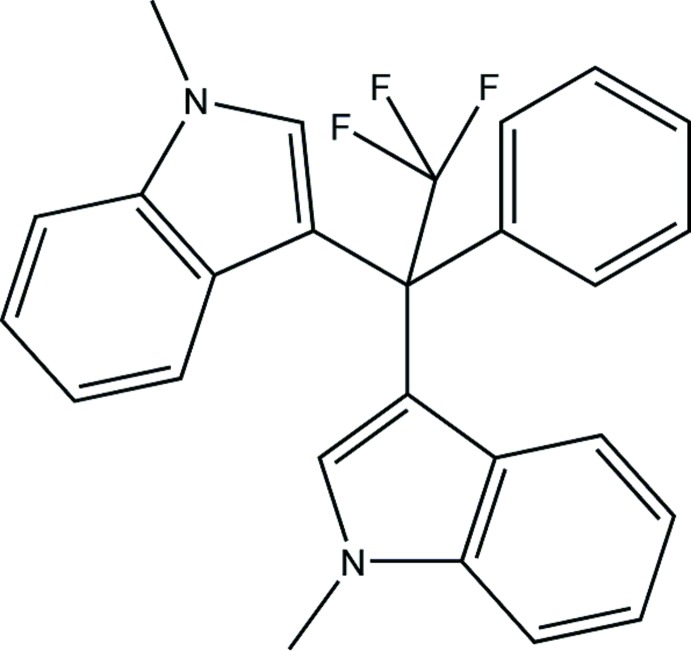


## Experimental   

### Crystal data  

C_26_H_21_F_3_N_2_
*M* *_r_* = 418.45
Monoclinic, 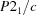

*a* = 10.0033 (3) Å
*b* = 12.9427 (3) Å
*c* = 16.2699 (7) Å
β = 102.571 (4)°
*V* = 2055.96 (12) Å^3^
*Z* = 4
Cu *K*α radiation
μ = 0.82 mm^−1^
*T* = 298 K
0.40 × 0.40 × 0.30 mm

### Data collection  

Bruker SMART diffractometer
Absorption correction: multi-scan (*SADABS*; Bruker, 2002 ▶) *T*_min_ = 0.736, *T*_max_ = 0.792
19108 measured reflections
3404 independent reflections
2777 reflections with *I* > 2σ(*I*)
*R*_int_ = 0.031

### Refinement  

*R*[*F* ^2^ > 2σ(*F* ^2^)] = 0.047
*wR*(*F* ^2^) = 0.153
*S* = 1.13
3404 reflections
282 parameters
6 restraints
H-atom parameters constrained
Δρ_max_ = 0.23 e Å^−3^
Δρ_min_ = −0.25 e Å^−3^

### 

Data collection: *SMART* (Bruker, 2002 ▶); cell refinement: *SAINT* (Bruker, 2002 ▶); data reduction: *SAINT*; program(s) used to solve structure: *SHELXTL* (Sheldrick, 2008 ▶); program(s) used to refine structure: *SHELXL97* (Sheldrick, 2008 ▶); molecular graphics: *DIAMOND* (Brandenburg, 2006 ▶); software used to prepare material for publication: *SHELXTL* and local programs.

## Supplementary Material

Crystal structure: contains datablock(s) I, New_Global_Publ_Block. DOI: 10.1107/S1600536814021916/hb7291sup1.cif

Structure factors: contains datablock(s) I. DOI: 10.1107/S1600536814021916/hb7291Isup2.hkl

Supporting information file. DOI: 10.1107/S1600536814021916/hb7291Isup4.doc

Supporting information file. DOI: 10.1107/S1600536814021916/hb7291Isup4.cml

**Fig. 1.**
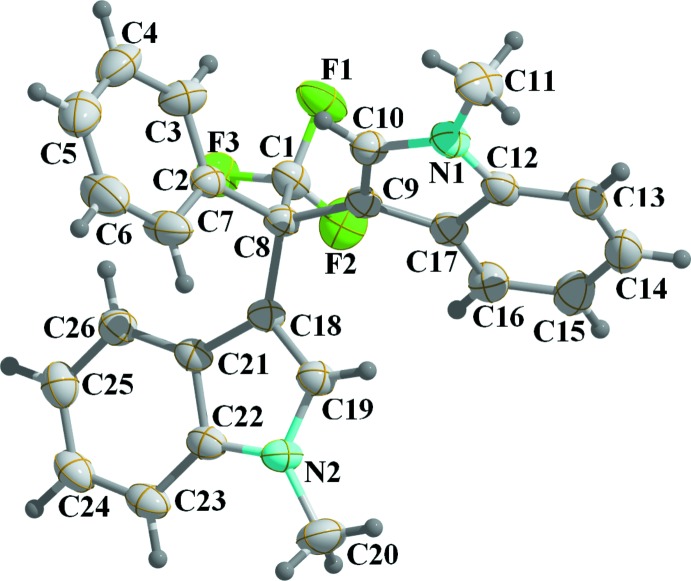
Plot of the title compound with the atom-numbering scheme. Displacement ellipsoids are represented at 40% probability levels.

. DOI: 10.1107/S1600536814021916/hb7291fig1.tif
Plot of the title compound with the atom-numbering scheme. Displacement ellipsoids are represented at 40% probability levels.

**Fig. 2.**
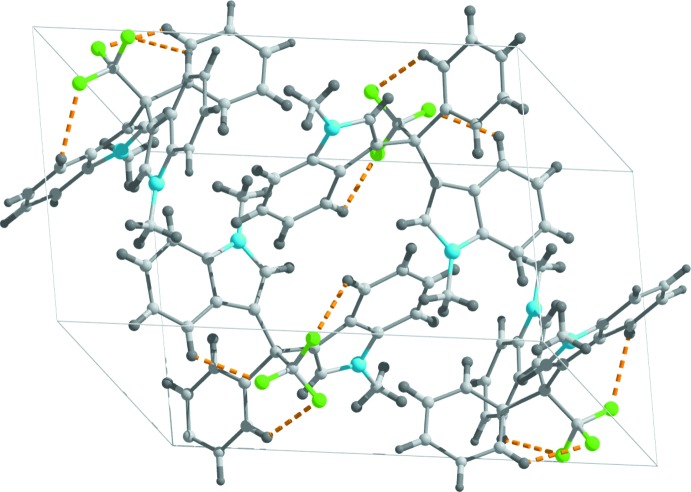
A crystal packing view of the title compound, showing the intramolecular C—H···F hydrogen bonds as dashed lines.

. DOI: 10.1107/S1600536814021916/hb7291fig2.tif
A crystal packing view of the title compound, showing the intra­molecular C—H⋯F hydrogen bonds as dashed lines.

CCDC reference: 1027554

Additional supporting information:  crystallographic information; 3D view; checkCIF report

## Figures and Tables

**Table 1:** Hydrogen-bond geometry (, )

*D*H*A*	*D*H	H*A*	*D* *A*	*D*H*A*
C3H3F1	0.93	2.32	2.969(3)	126
C16H16F3	0.93	2.51	3.029(2)	116
C26H26F2	0.93	2.42	2.989(2)	120

## supplementary crystallographic information

### S1. Introduction

The incorporation of tri­fluoro­methyl groups in active organic compounds may enhance the chemical, physical, and biological properties because the addition of tri­fluoro­methyl group can improve the metabolic stability and lipophilicity of various relevant cmopouns (Purser *et al.*, 2008). To date, a number of methods have been developed to install this functional group onto organic compounds, including palladium catalyzed (Shang *et al.*, 2014) and palladium mediated (Miura *et al.*, 2013) cross coupling reactions of aryl halides. In this context, we have tried to develop similar compounds containing tri­fluoro­methyl group. However, the unexpected title compound was obtained in one-step synthesis of reaction of a (2,2,2-tri­fluoro­ethyl) benzene with 1-methyl-1H-indole in such a condition of palladium-catalyzed. The important physiological activities of indole and its derivatives certainly have been also the subject of many studies (Lo *et al.* 2007).

The molecular structure of the title compound with atom numbering is shown is Fig. 1. All bond lengths and angles may be considered normal (Zhou *et al.*, 2011). All C substituents atoms adopt equatorial orientations. The dihedral angle for neighbouring indole ring and phenyl ring are 2.83 (2) and 0.4° respectively, which evidences the coplanarity between these groups.

In the crystal array three intra­molecular inter­actions C3—H3···F1 (2.969 Å), C16—H16···F3 (3.029 Å) and C26—H26···F2 (2.989 Å) of type hydrogen bonds are observed, and in the crystal packing inter­molecular contacts of non-classical hydrogen bonds are observed growing along the a, b and c axes, resulting in a complex supra­molecular array (Fig. 2).

### S2.1. Synthesis and crystallization

(2,2,2-tri­fluoro­ethyl) benzene (160 mg, 1.0 mmol) and PdCl_2_ (10mg) were added to a stirred solution of 1-methyl-1*H*-indole (393 mg, 3 mmol) in DMF (20 mL). After being refluxed at 373 K for 10 h, the mixture was dissolved in CH_2_Cl_2_, washed with saturated sodium bicarbonate solution (10 mL) and the organic layer was separated, dried over magnesium sulfate. Colourless blocks were prepared by slow evaporation of a solution of the title compound (24 mg) in CH_2_Cl_2_ (15 ml) and CH_3_OH (5 ml) at room temperature (yield 10%).

### S2.2. Refinement

Hydrogen atoms were clearly identified in difference syntheses, refined at idealized positions riding on the carbon atoms with isotropic displacement parameters Uiso(H) = 1.2Ueq(C) and C—H = 0.95–0.99 °.

### Crystal data

**Table:**

C_26_H_21_F_3_N_2_	*F*(000) = 872
*M**_r_* = 418.45	*D*_x_ = 1.352 Mg m^−^^3^
Monoclinic, *P*2_1_/*c*	Cu *K*α radiation, λ = 1.54178 Å
*a* = 10.0033 (3) Å	Cell parameters from 7360 reflections
*b* = 12.9427 (3) Å	θ = 4.4–72.2°
*c* = 16.2699 (7) Å	µ = 0.82 mm^−^^1^
β = 102.571 (4)°	*T* = 298 K
*V* = 2055.96 (12) Å^3^	Block, colourless
*Z* = 4	0.40 × 0.40 × 0.30 mm

### Data collection

**Table:**

Bruker SMART diffractometer	3404 independent reflections
Radiation source: fine-focus sealed tube	2777 reflections with *I* > 2σ(*I*)
Graphite monochromator	*R*_int_ = 0.031
Detector resolution: 16.0356 pixels mm^-1^	θ_max_ = 64.0°, θ_min_ = 4.4°
ω scans	*h* = −11→11
Absorption correction: multi-scan (*SADABS*; Bruker, 2002)	*k* = −15→15
*T*_min_ = 0.736, *T*_max_ = 0.792	*l* = −18→14
19108 measured reflections	

### Refinement

**Table:**

Refinement on *F*^2^	Primary atom site location: structure-invariant direct methods
Least-squares matrix: full	Secondary atom site location: difference Fourier map
*R*[*F*^2^ > 2σ(*F*^2^)] = 0.047	Hydrogen site location: inferred from neighbouring sites
*wR*(*F*^2^) = 0.153	H-atom parameters constrained
*S* = 1.13	*w* = 1/[σ^2^(*F*_o_^2^) + (0.1*P*)^2^] where *P* = (*F*_o_^2^ + 2*F*_c_^2^)/3
3404 reflections	(Δ/σ)_max_ < 0.001
282 parameters	Δρ_max_ = 0.23 e Å^−^^3^
6 restraints	Δρ_min_ = −0.25 e Å^−^^3^

### Special details

**Table:**

Geometry. All e.s.d.'s (except the e.s.d. in the dihedral angle between two l.s. planes) are estimated using the full covariance matrix. The cell e.s.d.'s are taken into account individually in the estimation of e.s.d.'s in distances, angles and torsion angles; correlations between e.s.d.'s in cell parameters are only used when they are defined by crystal symmetry. An approximate (isotropic) treatment of cell e.s.d.'s is used for estimating e.s.d.'s involving l.s. planes.
Refinement. Refinement of *F*^2^ against ALL reflections. The weighted *R*-factor *wR* and goodness of fit *S* are based on *F*^2^, conventional *R*-factors *R* are based on *F*, with *F* set to zero for negative *F*^2^. The threshold expression of *F*^2^ > σ(*F*^2^) is used only for calculating *R*-factors(gt) *etc*. and is not relevant to the choice of reflections for refinement. *R*-factors based on *F*^2^ are statistically about twice as large as those based on *F*, and *R*- factors based on ALL data will be even larger.

### Fractional atomic coordinates and isotropic or equivalent isotropic displacement parameters (Å^2^)

**Table:**

	*x*	*y*	*z*	*U*_iso_*/*U*_eq_	
C1	0.8971 (2)	0.93480 (14)	0.14490 (13)	0.0485 (5)	
C2	0.90591 (17)	0.81813 (12)	0.27157 (12)	0.0416 (4)	
C3	1.0436 (2)	0.79615 (15)	0.27931 (16)	0.0585 (6)	
H3	1.0866	0.8132	0.2359	0.070*	
C4	1.1181 (2)	0.74862 (17)	0.35173 (18)	0.0725 (6)	
H4	1.2106	0.7347	0.3563	0.087*	
C5	1.0571 (3)	0.72209 (18)	0.41642 (17)	0.0746 (7)	
H5	1.1074	0.6908	0.4648	0.090*	
C6	0.9224 (3)	0.7424 (2)	0.40857 (16)	0.0842 (8)	
H6	0.8796	0.7243	0.4518	0.101*	
C7	0.8474 (2)	0.78956 (18)	0.33715 (14)	0.0653 (6)	
H7	0.7548	0.8024	0.3333	0.078*	
C8	0.81404 (17)	0.86376 (12)	0.19119 (11)	0.0391 (4)	
C9	0.75931 (17)	0.77574 (12)	0.13113 (11)	0.0396 (4)	
C10	0.80448 (18)	0.67582 (13)	0.14276 (13)	0.0439 (5)	
H10	0.8679	0.6525	0.1896	0.053*	
C11	0.7736 (3)	0.50593 (14)	0.06680 (18)	0.0735 (7)	
H11A	0.8341	0.4812	0.1170	0.110*	
H11B	0.8162	0.4975	0.0198	0.110*	
H11C	0.6897	0.4673	0.0571	0.110*	
C12	0.65732 (18)	0.67488 (14)	0.01879 (13)	0.0470 (5)	
C13	0.5755 (2)	0.64716 (18)	−0.05864 (14)	0.0593 (6)	
H13	0.5734	0.5794	−0.0780	0.071*	
C14	0.4989 (2)	0.7221 (2)	−0.10526 (15)	0.0660 (6)	
H14	0.4439	0.7053	−0.1573	0.079*	
C15	0.5014 (2)	0.82402 (19)	−0.07616 (14)	0.0629 (6)	
H15	0.4470	0.8737	−0.1088	0.075*	
C16	0.5830 (2)	0.85165 (16)	−0.00007 (13)	0.0526 (5)	
H16	0.5845	0.9198	0.0181	0.063*	
C17	0.66373 (17)	0.77767 (13)	0.04998 (12)	0.0429 (4)	
C18	0.69956 (17)	0.92828 (12)	0.21498 (12)	0.0395 (4)	
C19	0.56319 (18)	0.91173 (13)	0.18853 (13)	0.0448 (5)	
H19	0.5246	0.8602	0.1509	0.054*	
C20	0.3410 (2)	0.98931 (19)	0.20577 (18)	0.0737 (7)	
H20A	0.3087	0.9838	0.2570	0.111*	
H20B	0.3023	0.9347	0.1681	0.111*	
H20C	0.3140	1.0548	0.1797	0.111*	
C21	0.71382 (19)	1.01305 (12)	0.27379 (11)	0.0400 (4)	
C22	0.57992 (19)	1.04430 (13)	0.27690 (12)	0.0432 (5)	
C23	0.5550 (2)	1.12422 (14)	0.32890 (13)	0.0545 (5)	
H23	0.4661	1.1446	0.3296	0.065*	
C24	0.6655 (2)	1.17181 (15)	0.37903 (14)	0.0597 (6)	
H24	0.6514	1.2253	0.4144	0.072*	
C25	0.7983 (2)	1.14148 (15)	0.37790 (13)	0.0588 (6)	
H25	0.8716	1.1750	0.4127	0.071*	
C26	0.8237 (2)	1.06329 (14)	0.32659 (13)	0.0495 (5)	
H26	0.9133	1.0438	0.3269	0.059*	
F1	0.98159 (12)	0.88259 (9)	0.10610 (8)	0.0695 (4)	
F2	0.97670 (12)	1.00240 (8)	0.19548 (8)	0.0601 (4)	
F3	0.81633 (14)	0.99051 (9)	0.08467 (8)	0.0687 (4)	
N1	0.74426 (16)	0.61530 (11)	0.07676 (11)	0.0496 (4)	
N2	0.48958 (16)	0.98118 (12)	0.22466 (11)	0.0487 (4)	

### Atomic displacement parameters (Å^2^)

**Table:**

	*U*^11^	*U*^22^	*U*^33^	*U*^12^	*U*^13^	*U*^23^
C1	0.0517 (11)	0.0426 (10)	0.0555 (13)	−0.0087 (8)	0.0209 (10)	−0.0075 (9)
C2	0.0391 (9)	0.0345 (8)	0.0515 (11)	0.0010 (7)	0.0105 (8)	−0.0099 (8)
C3	0.0424 (11)	0.0514 (11)	0.0826 (16)	0.0015 (9)	0.0154 (11)	0.0027 (11)
C4	0.0506 (12)	0.0616 (12)	0.0940 (16)	0.0091 (9)	−0.0089 (11)	−0.0052 (12)
C5	0.0772 (14)	0.0613 (12)	0.0745 (15)	0.0202 (11)	−0.0074 (12)	−0.0103 (11)
C6	0.109 (2)	0.0920 (18)	0.0557 (15)	0.0494 (16)	0.0258 (14)	0.0135 (14)
C7	0.0639 (13)	0.0758 (14)	0.0626 (14)	0.0301 (11)	0.0278 (12)	0.0159 (12)
C8	0.0380 (9)	0.0352 (8)	0.0462 (11)	−0.0019 (7)	0.0138 (8)	−0.0044 (8)
C9	0.0373 (9)	0.0374 (8)	0.0466 (11)	−0.0033 (7)	0.0148 (8)	−0.0046 (8)
C10	0.0398 (9)	0.0407 (9)	0.0527 (11)	−0.0009 (7)	0.0130 (8)	−0.0053 (8)
C11	0.0742 (16)	0.0415 (11)	0.104 (2)	−0.0002 (10)	0.0176 (15)	−0.0232 (12)
C12	0.0421 (10)	0.0492 (10)	0.0544 (12)	−0.0077 (8)	0.0207 (9)	−0.0114 (9)
C13	0.0516 (12)	0.0693 (13)	0.0602 (14)	−0.0142 (10)	0.0193 (11)	−0.0225 (12)
C14	0.0522 (13)	0.0952 (17)	0.0506 (13)	−0.0138 (12)	0.0114 (10)	−0.0152 (13)
C15	0.0555 (13)	0.0796 (15)	0.0523 (13)	−0.0036 (11)	0.0088 (11)	0.0079 (12)
C16	0.0546 (12)	0.0522 (11)	0.0520 (12)	−0.0021 (9)	0.0139 (10)	0.0031 (9)
C17	0.0397 (9)	0.0451 (9)	0.0471 (11)	−0.0053 (7)	0.0160 (8)	−0.0024 (8)
C18	0.0379 (9)	0.0339 (8)	0.0479 (11)	0.0002 (7)	0.0122 (8)	−0.0003 (7)
C19	0.0439 (11)	0.0394 (9)	0.0536 (12)	−0.0021 (7)	0.0158 (9)	−0.0043 (8)
C20	0.0411 (12)	0.0856 (16)	0.0958 (18)	0.0071 (10)	0.0178 (12)	−0.0080 (14)
C21	0.0449 (10)	0.0336 (8)	0.0438 (11)	0.0030 (7)	0.0149 (8)	0.0024 (8)
C22	0.0486 (11)	0.0378 (9)	0.0458 (11)	0.0077 (8)	0.0158 (9)	0.0063 (8)
C23	0.0634 (13)	0.0467 (10)	0.0577 (13)	0.0172 (9)	0.0229 (11)	0.0046 (10)
C24	0.0797 (15)	0.0457 (11)	0.0567 (13)	0.0118 (10)	0.0215 (12)	−0.0083 (10)
C25	0.0703 (14)	0.0513 (11)	0.0533 (13)	−0.0043 (10)	0.0100 (11)	−0.0078 (10)
C26	0.0502 (11)	0.0474 (10)	0.0521 (12)	−0.0005 (8)	0.0136 (10)	−0.0043 (9)
F1	0.0729 (9)	0.0667 (7)	0.0836 (9)	−0.0156 (6)	0.0494 (8)	−0.0168 (7)
F2	0.0601 (8)	0.0503 (6)	0.0739 (8)	−0.0207 (5)	0.0237 (6)	−0.0113 (6)
F3	0.0744 (9)	0.0618 (7)	0.0694 (9)	−0.0140 (6)	0.0146 (7)	0.0183 (6)
N1	0.0489 (9)	0.0375 (8)	0.0639 (11)	−0.0034 (7)	0.0161 (8)	−0.0134 (8)
N2	0.0383 (9)	0.0505 (9)	0.0599 (11)	0.0047 (7)	0.0162 (8)	−0.0011 (8)

### Geometric parameters (Å, º)

**Table:**

C1—F3	1.337 (2)	C12—C17	1.420 (2)
C1—F2	1.338 (2)	C13—C14	1.360 (3)
C1—F1	1.342 (2)	C13—H13	0.9300
C1—C8	1.541 (2)	C14—C15	1.400 (3)
C2—C7	1.375 (3)	C14—H14	0.9300
C2—C3	1.385 (3)	C15—C16	1.374 (3)
C2—C8	1.543 (3)	C15—H15	0.9300
C3—C4	1.393 (3)	C16—C17	1.395 (3)
C3—H3	0.9300	C16—H16	0.9300
C4—C5	1.370 (4)	C18—C19	1.355 (2)
C4—H4	0.9300	C18—C21	1.442 (2)
C5—C6	1.351 (4)	C19—N2	1.373 (2)
C5—H5	0.9300	C19—H19	0.9300
C6—C7	1.381 (3)	C20—N2	1.454 (3)
C6—H6	0.9300	C20—H20A	0.9600
C7—H7	0.9300	C20—H20B	0.9600
C8—C9	1.523 (2)	C20—H20C	0.9600
C8—C18	1.533 (2)	C21—C26	1.400 (3)
C9—C10	1.369 (2)	C21—C22	1.410 (3)
C9—C17	1.452 (3)	C22—N2	1.368 (3)
C10—N1	1.359 (2)	C22—C23	1.393 (3)
C10—H10	0.9300	C23—C24	1.369 (3)
C11—N1	1.462 (2)	C23—H23	0.9300
C11—H11A	0.9600	C24—C25	1.390 (3)
C11—H11B	0.9600	C24—H24	0.9300
C11—H11C	0.9600	C25—C26	1.370 (3)
C12—N1	1.372 (3)	C25—H25	0.9300
C12—C13	1.392 (3)	C26—H26	0.9300
			
F3—C1—F2	106.37 (15)	C13—C14—C15	121.1 (2)
F3—C1—F1	105.59 (15)	C13—C14—H14	119.4
F2—C1—F1	105.51 (15)	C15—C14—H14	119.4
F3—C1—C8	112.05 (15)	C16—C15—C14	120.9 (2)
F2—C1—C8	113.65 (16)	C16—C15—H15	119.6
F1—C1—C8	113.03 (14)	C14—C15—H15	119.6
C7—C2—C3	117.21 (19)	C15—C16—C17	120.2 (2)
C7—C2—C8	119.02 (16)	C15—C16—H16	119.9
C3—C2—C8	123.56 (17)	C17—C16—H16	119.9
C2—C3—C4	120.4 (2)	C16—C17—C12	117.33 (18)
C2—C3—H3	119.8	C16—C17—C9	136.51 (17)
C4—C3—H3	119.8	C12—C17—C9	106.16 (16)
C5—C4—C3	121.0 (2)	C19—C18—C21	106.15 (15)
C5—C4—H4	119.5	C19—C18—C8	126.10 (15)
C3—C4—H4	119.5	C21—C18—C8	127.61 (15)
C6—C5—C4	118.7 (2)	C18—C19—N2	110.93 (16)
C6—C5—H5	120.6	C18—C19—H19	124.5
C4—C5—H5	120.6	N2—C19—H19	124.5
C5—C6—C7	120.9 (2)	N2—C20—H20A	109.5
C5—C6—H6	119.6	N2—C20—H20B	109.5
C7—C6—H6	119.6	H20A—C20—H20B	109.5
C2—C7—C6	121.8 (2)	N2—C20—H20C	109.5
C2—C7—H7	119.1	H20A—C20—H20C	109.5
C6—C7—H7	119.1	H20B—C20—H20C	109.5
C9—C8—C18	112.66 (14)	C26—C21—C22	118.04 (16)
C9—C8—C1	106.82 (14)	C26—C21—C18	135.37 (16)
C18—C8—C1	108.08 (13)	C22—C21—C18	106.52 (16)
C9—C8—C2	108.79 (13)	N2—C22—C23	129.75 (18)
C18—C8—C2	109.53 (14)	N2—C22—C21	108.09 (15)
C1—C8—C2	110.94 (15)	C23—C22—C21	122.12 (19)
C10—C9—C17	105.90 (15)	C24—C23—C22	117.91 (19)
C10—C9—C8	123.78 (17)	C24—C23—H23	121.0
C17—C9—C8	130.10 (15)	C22—C23—H23	121.0
N1—C10—C9	111.03 (17)	C23—C24—C25	121.02 (18)
N1—C10—H10	124.5	C23—C24—H24	119.5
C9—C10—H10	124.5	C25—C24—H24	119.5
N1—C11—H11A	109.5	C26—C25—C24	121.4 (2)
N1—C11—H11B	109.5	C26—C25—H25	119.3
H11A—C11—H11B	109.5	C24—C25—H25	119.3
N1—C11—H11C	109.5	C25—C26—C21	119.47 (19)
H11A—C11—H11C	109.5	C25—C26—H26	120.3
H11B—C11—H11C	109.5	C21—C26—H26	120.3
N1—C12—C13	129.57 (18)	C10—N1—C12	108.95 (15)
N1—C12—C17	107.95 (16)	C10—N1—C11	125.48 (18)
C13—C12—C17	122.5 (2)	C12—N1—C11	125.48 (18)
C14—C13—C12	118.0 (2)	C22—N2—C19	108.28 (16)
C14—C13—H13	121.0	C22—N2—C20	126.47 (16)
C12—C13—H13	121.0	C19—N2—C20	125.14 (18)
			
C7—C2—C3—C4	−1.0 (3)	C13—C12—C17—C9	−179.68 (16)
C8—C2—C3—C4	−175.69 (17)	C10—C9—C17—C16	−179.4 (2)
C2—C3—C4—C5	0.4 (3)	C8—C9—C17—C16	−4.6 (3)
C3—C4—C5—C6	0.4 (4)	C10—C9—C17—C12	0.87 (18)
C4—C5—C6—C7	−0.5 (4)	C8—C9—C17—C12	175.62 (16)
C3—C2—C7—C6	1.0 (3)	C9—C8—C18—C19	0.9 (2)
C8—C2—C7—C6	175.9 (2)	C1—C8—C18—C19	118.7 (2)
C5—C6—C7—C2	−0.2 (4)	C2—C8—C18—C19	−120.32 (19)
F3—C1—C8—C9	73.56 (17)	C9—C8—C18—C21	176.09 (16)
F2—C1—C8—C9	−165.82 (15)	C1—C8—C18—C21	−66.1 (2)
F1—C1—C8—C9	−45.6 (2)	C2—C8—C18—C21	54.9 (2)
F3—C1—C8—C18	−47.91 (19)	C21—C18—C19—N2	1.4 (2)
F2—C1—C8—C18	72.7 (2)	C8—C18—C19—N2	177.41 (16)
F1—C1—C8—C18	−167.07 (16)	C19—C18—C21—C26	175.2 (2)
F3—C1—C8—C2	−168.01 (13)	C8—C18—C21—C26	−0.7 (3)
F2—C1—C8—C2	−47.39 (19)	C19—C18—C21—C22	−1.6 (2)
F1—C1—C8—C2	72.83 (19)	C8—C18—C21—C22	−177.52 (16)
C7—C2—C8—C9	−89.0 (2)	C26—C21—C22—N2	−176.26 (15)
C3—C2—C8—C9	85.5 (2)	C18—C21—C22—N2	1.21 (19)
C7—C2—C8—C18	34.5 (2)	C26—C21—C22—C23	1.5 (3)
C3—C2—C8—C18	−150.95 (16)	C18—C21—C22—C23	178.98 (16)
C7—C2—C8—C1	153.72 (18)	N2—C22—C23—C24	176.23 (19)
C3—C2—C8—C1	−31.7 (2)	C21—C22—C23—C24	−1.0 (3)
C18—C8—C9—C10	−131.40 (17)	C22—C23—C24—C25	0.1 (3)
C1—C8—C9—C10	110.07 (19)	C23—C24—C25—C26	0.2 (3)
C2—C8—C9—C10	−9.8 (2)	C24—C25—C26—C21	0.3 (3)
C18—C8—C9—C17	54.7 (2)	C22—C21—C26—C25	−1.1 (3)
C1—C8—C9—C17	−63.9 (2)	C18—C21—C26—C25	−177.68 (18)
C2—C8—C9—C17	176.33 (16)	C9—C10—N1—C12	0.4 (2)
C17—C9—C10—N1	−0.82 (19)	C9—C10—N1—C11	177.06 (19)
C8—C9—C10—N1	−175.99 (15)	C13—C12—N1—C10	179.10 (18)
N1—C12—C13—C14	−179.21 (18)	C17—C12—N1—C10	0.1 (2)
C17—C12—C13—C14	−0.4 (3)	C13—C12—N1—C11	2.5 (3)
C12—C13—C14—C15	−0.3 (3)	C17—C12—N1—C11	−176.47 (19)
C13—C14—C15—C16	1.0 (3)	C23—C22—N2—C19	−177.94 (18)
C14—C15—C16—C17	−0.8 (3)	C21—C22—N2—C19	−0.4 (2)
C15—C16—C17—C12	0.1 (3)	C23—C22—N2—C20	5.7 (3)
C15—C16—C17—C9	−179.65 (19)	C21—C22—N2—C20	−176.8 (2)
N1—C12—C17—C16	179.56 (15)	C18—C19—N2—C22	−0.6 (2)
C13—C12—C17—C16	0.5 (3)	C18—C19—N2—C20	175.8 (2)
N1—C12—C17—C9	−0.62 (18)		

### Hydrogen-bond geometry (Å, º)

**Table:**

*D*—H···*A*	*D*—H	H···*A*	*D*···*A*	*D*—H···*A*
C3—H3···F1	0.93	2.32	2.969 (3)	126
C16—H16···F3	0.93	2.51	3.029 (2)	116
C26—H26···F2	0.93	2.42	2.989 (2)	120
