# Supplementary material for: Crystal structure of 1-methyl-3-[2,2,2-tri­fluoro-1-(1-methyl-1H-indol-3-yl)-1-phenyl­eth­yl]-1H-indole
Source: Acta Crystallogr Sect E Struct Rep Online. 2014 Oct 11;70(Pt 11):o1156. doi: 10.1107/S1600536814021916 (PMC4257249; doi:10.1107/S1600536814021916)
Supplement: Supplementary file 3 [file e-70-o1156-Isup4.doc]

Crystal structure of 3-(2,2,2-trifluoro-1-(1-methyl-1*H*-indol-3-yl)-1-phenylethyl)-1-methyl-1*H*-indole

Xian-Rong Liu, Yan-Ling Zhou

S1.Introduction

The incorporation of trifluoromethyl groups in active organic compounds may enhance the chemical, physical, and biological properties because the addition of trifluoromethyl group can improve the metabolic stability and lipophilicity of various relevant cmopouns (Purser *et al*. 2008). To date, a number of methods have been developed to install this functional group onto organic compounds, including palladium catalyzed (Shang *et al*. 2014) and palladium mediated (Miura *et al*. 2013) cross coupling reactions of aryl halides. In this context, we have tried to develop similar compounds containing trifluoromethyl group. However, the unexpected title compound was obtained in one-step synthesis of reaction of a (2,2,2-trifluoroethyl) benzene with 1-methyl-1H-indole in such a condition of palladium-catalyzed. The important physiological activities of indole and its derivatives certainly have been also the subject of many studies (Lo *et al*. 2007).

The molecular structure of the title compound with atom numbering is shown is Fig. 1. All bond lengths and angles may be considered normal (Zhou *et al*. 2011). All C substituents atoms adopt equatorial orientations. The dihedral angle for neighbouring indole ring and phenyl ring are 2.83(2)° and 0.4° respectively, which evidences the coplanarity between these groups. In the crystal array three intramolecular interaction C3-H3···F1 (2.969 Å), C16-H16···F3 (3.029 Å) and C26-H26···F2 (2.989 Å) of type hydrogen bonds are observed, and in the crystal packing intermolecular contacts of non-classical hydrogen bonds are observed growing along the *a,* *b* and *c* axes, resulting in a complex supramolecular array (Fig. 2).

S2. Refinement

Crystal data, data collection and structure refinement details are summarized in Table 1. Hydrogen atoms were clearly identified in difference syntheses, refined at idealized positions riding on the carbon atoms with isotropic displacement parameters U_iso_(H) = 1.2U_eq_(C) and C–H 0.95–0.99 Å.

S3. Preparation

(2,2,2-trifluoroethyl) benzene (160 mg, 1.0 mmol) and PdCl_2_ (10mg) were added to a stirred solution of 1-methyl-1*H*-indole (393 mg, 3 mmol) in DMF (20 mL). After being refluxed at 373 K for 10 h, the mixture was dissolved in CH_2_Cl_2_, washed with saturated sodium bicarbonate solution (10 mL) and the organic layer was separated, dried over magnesium sulfate. Single crystals suitable for X-ray diffraction were prepared by slow evaporation of a solution of the title compound (24 mg) in CH_2_Cl_2_ (15 mL) and CH_3_OH (5 ml) at room temperature (yield 10%).

Figure 1 Plot of the title compound with the atom-numbering scheme. Displacement ellipsoids are represented at 40% probability levels.

Figure 2 A crystal packing view of the title compound, only showing the classical C-H···F intramolecular hydrogen bond.
