# Supplementary figures and images for: Crystal structure of 1-methyl-3-[2,2,2-tri­fluoro-1-(1-methyl-1H-indol-3-yl)-1-phenyl­eth­yl]-1H-indole
Source: Acta Crystallogr Sect E Struct Rep Online. 2014 Oct 11;70(Pt 11):o1156. doi: 10.1107/S1600536814021916 (PMC4257249; doi:10.1107/S1600536814021916)

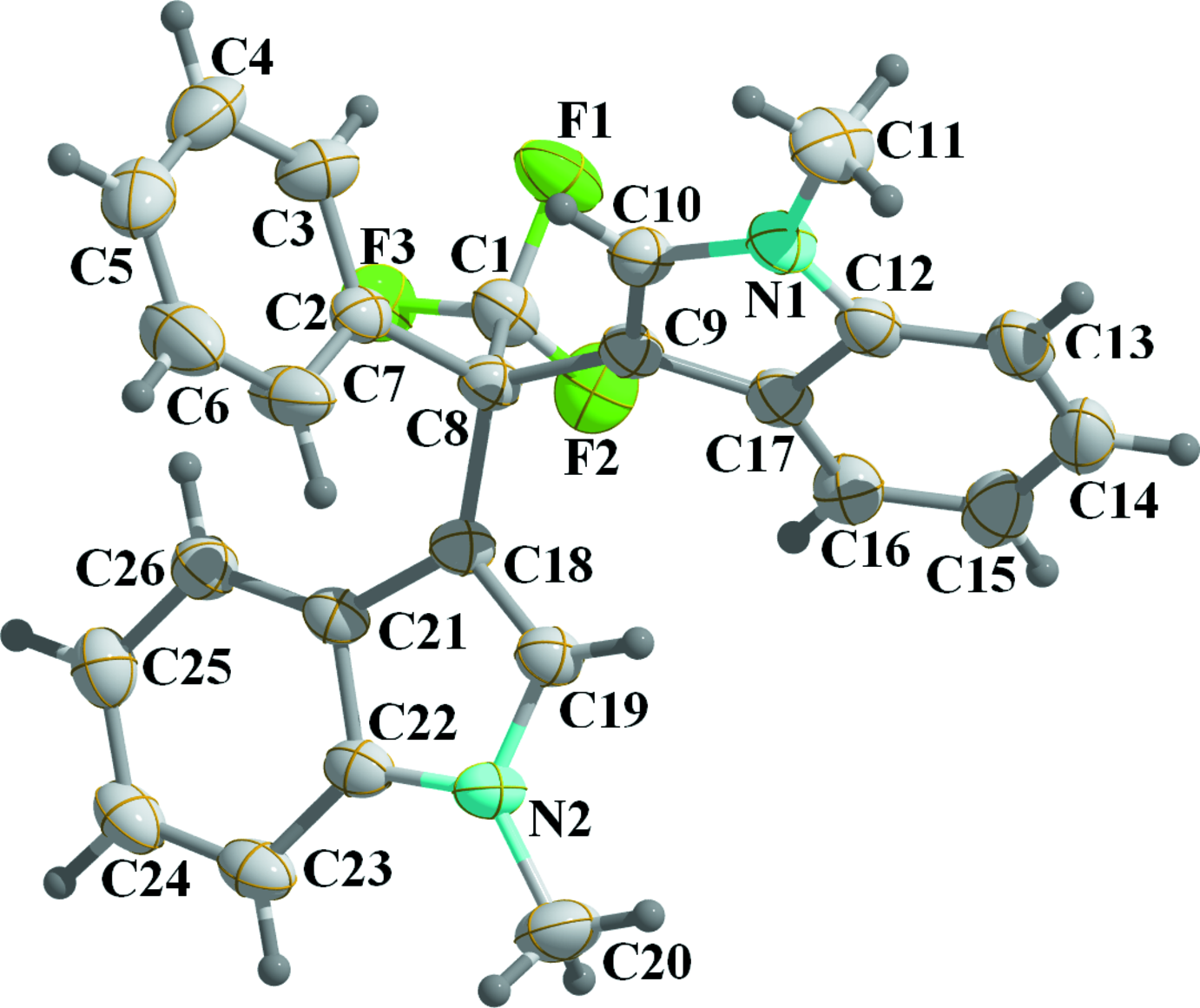

Supplement: Supplementary file 5 [file e-70-o1156-fig1.tif]

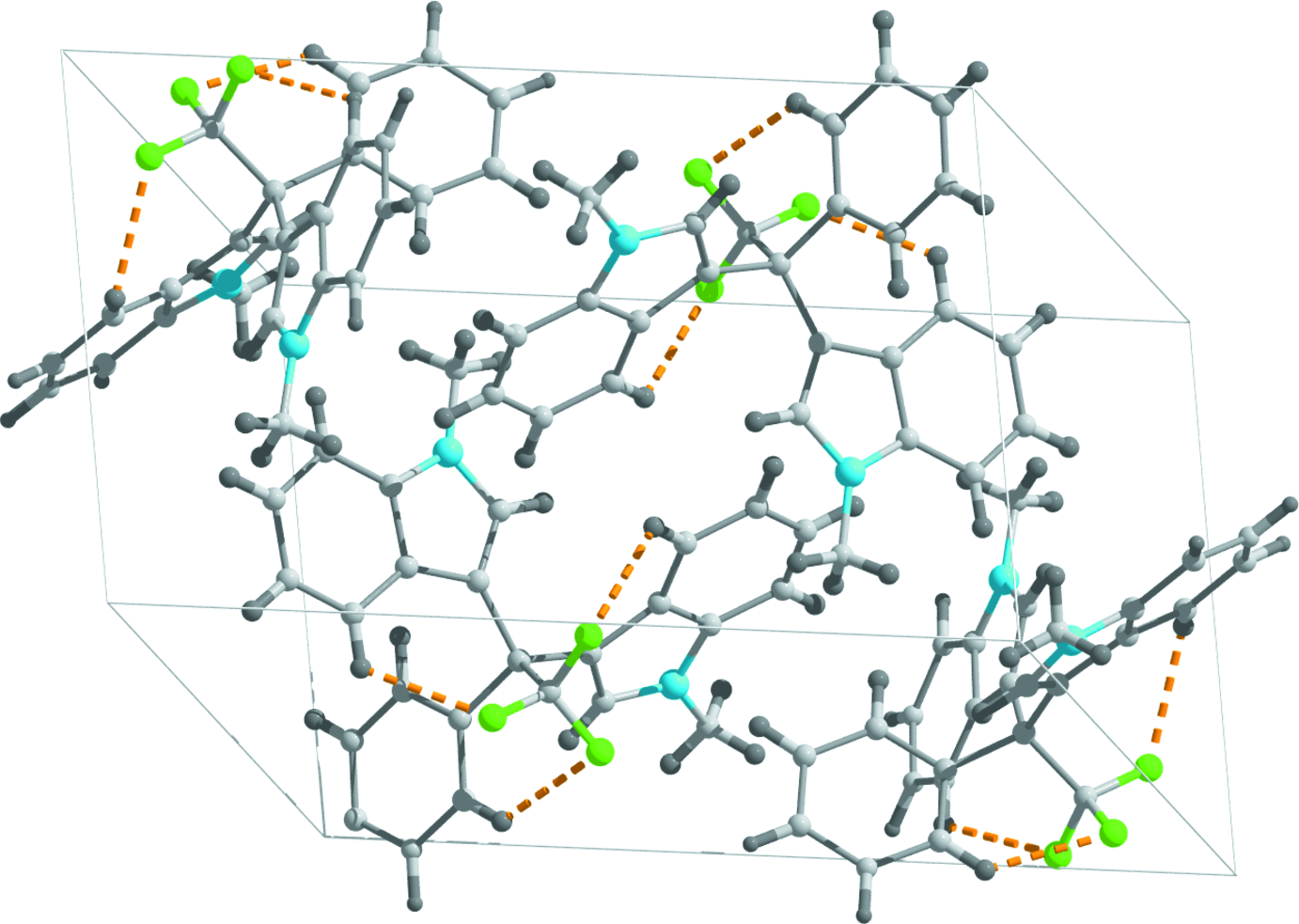

Supplement: Supplementary file 6 [file e-70-o1156-fig2.tif]
